# Information Technology-Enhanced Telehealth Consultations Reduce Preoperative Evaluation Center Visits in a Bariatric Surgery Population

**DOI:** 10.3390/healthcare11030309

**Published:** 2023-01-19

**Authors:** Jill E. Zafar, Kathleen T. Chan, Lori J. Ryder, Andrew J. Duffy, Feng Dai, Zyad J. Carr, Jean G. Charchaflieh

**Affiliations:** 1 Yale School of Medicine, Department of Anesthesiology, New Haven, CT 06510, USA; 2 Yale New Haven Hospital, New Haven, CT 06510, USA; 3 Yale School of Medicine, Department of General Surgery, Division of Yale Bariatric/Gastrointestinal Surgery, Temple Medical Center, New Haven, CT 06510, USA

**Keywords:** preoperative evaluation, anesthesia, bariatric surgery, perioperative medicine, information technology

## Abstract

**Background**: Preoperative patient evaluation and optimization in a preoperative evaluation center (PEC) has been shown to improve operating room (OR) efficiency and patient care. However, performing preoperative evaluation on all patients scheduled for surgery or procedure would be time- and resource-consuming. Therefore, appropriate patient selection for evaluation at PECs is one aspect of improving PEC efficiency. In this study, we evaluate the effect of an enhanced preoperative evaluation process (PEP), utilizing a nursing triage phone call and information technology (IT) optimizations, on PEC efficiency and the quality of care in bariatric surgery patients. We hypothesized that, compared to a traditional PEP, the enhanced PEP would improve PEC efficiency without a negative impact on quality. **Methods**: The study was a retrospective cohort analysis of 1550 patients from January 2014 to March 2017 at a large, tertiary care academic health system. The study was a before/after comparison that compared the enhanced PEP model to the traditional PEP model. The primary outcome was the efficiency of the PEC, which was measured by the reduction of in-person patient visits at the PEC. The secondary outcome was the quality of care, which was measured by delays, cancellations, and the need for additional testing on the day of surgery (DOS). **Results**: The enhanced PEP improved the primary outcome of efficiency, as evident by an 80% decrease in in-person patient visits to the PEC. There was no reduction in the secondary outcome of the quality of care as measured by delays, cancellations, or the need for additional testing on the DOS. The implementation of the enhanced PEP did not result in increased costs or resource utilization. **Conclusions**: The enhanced PEP in a multi-disciplinary preoperative process can improve the efficiency of PEC for bariatric surgery patients without any decrease in the quality of care. The enhanced PEP process can be implemented without an increase in resource utilization and can be particularly useful during the COVID-19 pandemic.

## 1. Introduction

The practice of preoperative evaluation of patients at a preoperative evaluation center (PEC) was first introduced in the United States in the 1950s. For decades, the practice of preoperative evaluation of patients in PECs was implemented without being scientifically evaluated regarding its impact on patient care and other variables. In 1996, Fischer published one of the earliest reports that demonstrated the value of a PEC in a teaching hospital in reducing both same-day case cancellation and unnecessary testing [1]. Subsequent studies have demonstrated the value of PEC in reducing both case delay and case cancellation on the day of surgery (DOS) [2]. Studies that have implemented cost-of-care analysis have demonstrated that PEC can be effective in reducing the overall cost of perioperative care [1,2,3]. More recently, anesthesiologist-led preoperative evaluations in a PEC were shown to reduce in-hospital mortality [4]. Although PECs have been rapidly adopted over the last fifty years in the United States, the ideal model remains unclear [5]

The predominant model is for PECs to be part of anesthesia practice in high-volume academic institutions or hospitals [1,6,7,8,9]. However, within PECs, a wide variety of operational variations exist in the areas of which patients are selected for PEC evaluation, personnel staffing for PECs, and the operational structure of PEC. For example, patient selection for evaluation in a PEC may occur using an exploratory phone call, an electronic questionnaire, or a questionnaire during the preoperative surgical visit [1,7,9]. Personnel staffing of PEC may include any combination of registered nurses (RNs), nurse practitioners (NPs), anesthesiology residents, anesthesiologists, or medical internists [1,4,5,6,7,10]. Most variations include either the presence of an on- or off-site anesthesiologist for advanced consultation. The most common operational model of a PEC consists of an in-person visit, usually upon surgical referral [1,7,10]

The lack of universal implementation of PEC may be due to the significant upfront cost, typically a sunk cost. Therefore, reducing the number of in-person PEC visits may offer an attractive cost incentive for its implementation [11]. Telephone preoperative assessments have been utilized in select PEC models but are usually reserved for healthier patients or low-risk surgeries [10,12,13,14]. In this retrospective pre-post study, we compared an enhanced preoperative evaluation process (PEP) through the use of IT (information technology) and a nursing triage system to a traditional PEC model in bariatric surgery patients.

Telehealth utilization has increased dramatically in the past decade, accelerated by the COVID-19 pandemic. Telehealth has been associated with increased access to care, increased patient satisfaction, decreased environmental impact, and reduced costs [15,16,17]. Telehealth has been used successfully for preoperative consultation in several surgical specialties, including bariatric surgery [18,19,20]. During the COVID-19 pandemic, the use of telehealth in bariatric surgery allowed for the safe continuation of elective surgery with increased access to care [21,22,23,24,25,26,27]. There have been no studies examining the preoperative anesthesia evaluation of patients using telehealth.

Our primary outcome was the efficiency of the enhanced PEP model as measured by the number of PEC visits in comparison to the traditional model in elective bariatric surgery patients. Our secondary outcomes evaluated the potential risks for the quality of care in the enhanced model, as measured by first-case start delays, cancellations, or the need for additional testing on the day of surgery (DOS), in comparison to the traditional model. We hypothesized that the enhanced PEC would provide increased efficiency without an increase in measured risk for the quality of care.

## 2. Methods

The study was conducted at Yale New Haven Health (YNHH), which is a tertiary academic medical system that includes several hospitals and ambulatory surgery centers (ASCs). Bariatric surgery at YNHH was performed at two hospitals with a combined bed capacity of 1541 beds and an annual surgical volume of 50,000 surgeries. The PEC at YNHH is an anesthesiologist-led center that collaborates with the perioperative nursing and surgery departments. The YNHH PEC is responsible for the evaluation and preoperative preparation of about 35,000 patients yearly, with a daily staff of 12 registered nurses (RNs), 5 nurse practitioners (NPs), 2 patient care assistants (PCAs), 3 clerical assistants (CAs), 2 anesthesiology residents, and an attending anesthesiologist.

### 2.1. Pre-Intervention: Traditional Model for Preoperative Screening

The traditional preoperative evaluation process was dependent on the surgical provider triaging their patients and referring them to the anesthesiologist-led PEC. The PEC provided guidelines for patient selection criteria to help guide the surgical decision-making process for referral. In addition, patients who were not referred to the PEC received a phone call from an RN 1–2 days prior to surgery and were given basic preoperative instructions regarding preoperative fasting and medication management. Patients referred to the PEC received a full preoperative anesthesia evaluation, indicating preoperative testing, consult evaluations, and preoperative instructions regarding preoperative fasting and medication management.

### 2.2. Post-Intervention: Telehealth-Integrated PEP Model for Preoperative Screening

**Figure 1:**
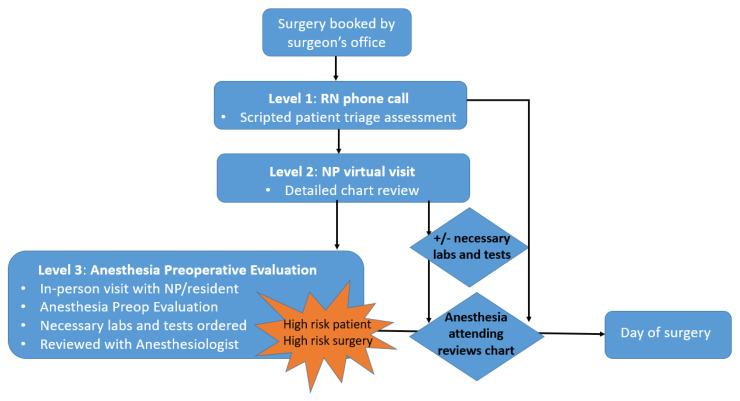
Preoperative evaluation center flowchart. The process starts with the surgeon scheduling the surgery. Level 1: the registered nurse (RN) calls the patient. The next phase of care is determined by a triage algorithm (Appendix B) and can include: (1) no further work-up needed, (2) additional preoperative testing, which could be arranged at a location convenient to the patient, (3) a chart review conducted by a nurse practitioner (NP), (4) an in-person visit to the PEC. All charts are reviewed by the anesthesiologist on the day before surgery.

Through a quality improvement initiative, the enhanced PEP screening process (Figure 1) was developed in addition to new preoperative testing guidelines (Appendix A). The process began with a scripted phone encounter with a triage nurse. Based on this encounter, the patient’s medical history was entered into the electronic health record (EHR). Then, the triage nurse used a triage algorithm integrated into the EHR to perform a stepwise decision-making process to determine patient allocation into one of the following four endpoints: (1) no further work-up needed (patient ready for surgery), (2) additional preoperative testing suggested, (3) chart review by an NP required, or (4) in-person visit to the PEC required (Appendix B). When an NP chart review was required, the NP reviewed the patient’s EHR to determine if any further testing, evaluations (consults), or in-person visits were needed prior to surgery. When an in-person PEC visit was required, the same format for the PEC visit was used during both periods of before and after the implementation of the enhanced PEP.

### 2.3. IT Optimization

The YNHH EHR system is supported by Epic Systems (Wisconsin, USA). The Epic system has many features that allowed for the creation and utilization of the enhanced PEP. The process began by creating worklists to support the workflow and ensure a high-reliability process. Customizable worklists were created and used by RNs, NPs, and Cas. All the worklists could be filtered by the date of surgery, patient name, surgeon, surgical service, required follow-up tasks, and PEC provider. The EHR triage tool utilized a Best Practice Advisory (BPA), embedded within Epic and developed with multi-disciplinary consensus and incorporated patient medication history, past medical, surgical, and social history. Another Epic optimization involved the implementation of an EHR module (Procedure Pass™), which provided a common place to file all necessary preoperative documentation. Clinical decision support was integrated into Procedure Pass. Using information about the patient’s age, scheduled surgery (current procedural terminology (CPT) code), medical, surgical, or social history, and medication list, Procedure Pass recommended preoperative testing requirements. In addition, institutional preoperative testing guidelines, specific surgical service requirements, and a surgical blood order schedule were integrated into Procedure Pass. If it was already performed, the RN would verify this step; otherwise, the RN would coordinate with the patient to have the testing performed. Procedure Pass was utilized as a common platform and facilitated communication among healthcare providers. A patient was deemed ready to proceed to their scheduled procedure when all Procedure Pass tasks were complete. A final optimization was the development of a call center with a single phone number to simplify interactions with patients.

### 2.4. Data Collection

This was a retrospective case–control study analyzing data from the Yale Bariatric/Gastrointestinal Surgery service at YNHH. No IRB approval was needed as the project was classified as a quality improvement study (Yale University School of Medicine Institutional Review Board, New Haven, CT, USA). The study encompassed 1550 patients over a three-year period, with each timeframe of pre- and post-intervention consisting of 19 months. The pre-intervention period was from 2 January 2014 to 31 July 2015 and yielded 810 patients. The post-intervention period was from 1 September 2015 to 31 March 2017 and yielded 740 patients. Data from 1–31 August 2015 were not included in the study as this month was the transition point between the old and new preoperative process. The exclusion criteria included actively hospitalized inpatients, add-on cases (scheduled within 24 h of surgery date), and patients less than 18 years old.

Bariatric surgery outpatients included in the study were scheduled with one of the following CPT codes: 43644 (laparoscopic gastric restrictive procedure with gastric bypass and Roux-en-Y gastroenterostomy), 43775 (laparoscopic gastric restrictive procedure; longitudinal gastrectomy), 43770 (laparoscopic gastric restrictive procedure with placement of adjustable restrictive device), 43771 (laparoscopic revision of adjustable gastric band component only), 43773 (laparoscopic removal and replacement of adjustable gastric band component only), 43774 (laparoscopic removal of adjustable gastric band and subcutaneous port components), and 43848 (open revision of gastric restrictive procedure for morbid obesity, other than adjustable gastric restrictive device).

Data were collected electronically and manually using a chart review. Information collected electronically from our EHR included the surgery date, surgeon of record, surgical service, scheduled surgical procedure and CPT code, scheduled start time, actual operating room start time, patient name, patient age, American Society of Anesthesiologists (ASA) Physical Status Classification, medical record number (MRN), surgical class (outpatient versus inpatient), add-on classification, DOS cancellations, cancellation reason, triage phone call result, and absence or presence of a PEC visit. The data that were manually extracted from the EHR included DOS testing and the reason for DOS cancellation.

### 2.5. Outcomes

To compare the efficacy of the telehealth-integrated model with the traditional model, we analyzed the following outcomes between groups: the number of patients with in-person visits to the PEC during the post-intervention period in comparison to the pre-intervention period, DOS additional testing, first-case start delays, and DOS cancellations. DOS testing was defined as a laboratory test, electrocardiogram (ECG), or chest X-ray (CXR) performed on the day of surgery prior to entry into the operating room. We excluded the following routine DOS preoperative labs: point-of-care blood glucose (BG) testing, point-of-care urine human chorionic gonadotropin (hCG) pregnancy testing, and prothrombin time (PT) and international randomization ratio (INR) for patients on warfarin. The collation of the DOS testing results was restricted to a 2-month duration during the pre-intervention period (June and July 2015) and a 2-month duration during the post-intervention period (February and March 2017). DOS cancellations were examined by an experienced perioperative physician by chart review to determine if the cancellation could or could not have been attributed to an incomplete preoperative evaluation. First-case surgeries were defined as any scheduled start time at or before 8:30 am. A delay was defined as entering the operating room any time after the scheduled start time.

### 2.6. Statistical Analysis

Patient demographics, clinical characteristics, and outcomes were summarized using the mean plus or minus the standard deviation (±SD) for continuous variables, and the number (n) and percentage (%) for categorical variables. Univariate analyses or simple statistical comparison of different variables between the pre-invention and the post-intervention groups was performed by a two-sample *t*-test or Fisher’s exact test, as appropriate. Multiple logistic regression analysis was performed to assess the association between the PEC requirement (Yes vs. No) and intervention groups (post-intervention vs. pre-intervention). All the statistical analyses were performed using SAS v9.4 (Cary, NC, USA), and a two-sided *p*-value of less than 0.05 was considered significant.

Based on the estimated proportion of in-person visits to the PEC during the pre-intervention period (70%), a sample size of 700 patients per group (pre- vs. post-intervention) would achieve 80% power to detect a small difference of 8% (equivalent to a small Cohen’s effect size of 0.15) using the two-sided Z test with the pooled variance targeted and a significance level of 0.05.

## 3. Results

Demographic information about the pre-intervention and post-intervention groups is described in Table 1. There was no difference between the two groups in the average age distribution (44.54 [±12.13] vs. 44.75 [±12.05], *p* = 0.72) or surgeries performed (CPT). The distribution of ASA classifications was different between the pre-intervention and post-intervention groups (*p* = 0.015). ASA classifications were grouped into two categories (ASA 1 and 2 vs. ASA 3 and 4) in the multiple regression analysis. There was a difference in ASA status between the groups (ASA 1–2: 214 patients (26.4%) pre-intervention versus 151 patients (20.4%) post-intervention; ASA 3–4: 596 patients (73.6%) pre-intervention vs. 589 patients (79.6%) post-intervention; (*p* = 0.015)).

The primary and secondary outcomes are presented in Table 2. Post-intervention, the preoperative process reduced PEC visits (15.4% vs. 75.9%, (*p* < 0.001)). Among the secondary outcomes, we found a 40% decrease in patient cost per preoperative encounter (USD 50.24 to USD 30.56 pre- and post-intervention respectively, *p* < 0.001). DOS cancellations (*p* = 0.84), DOS first-case start delays (*p* = 0.10), and DOS laboratory testing were unchanged between the groups, with the exception of a decrease in blood screening (type and screen laboratory investigation), which was explained by an institutional policy change during the study period. Post-intervention, an increase in DOS EKG orders was observed (*p* = 0.003). After adjusting for ASA status (ASA 1–2 vs. ASA 3–4), the odds of an in-person PEC visit requirement in the post-intervention group was lower than the pre-intervention group (odds ratio (OR) = 0.05; 95% confidence interval (CI) = 0.04–0.07; *p* < 0.001) (Table 3).

Furthermore, to assess whether higher acuity patients were seen more frequently with in-person visits to the PEC, we analyzed the percentage of patients in each ASA classification with PEC visits, but this did not reach significance (Table 4). In the pre-intervention group, an ASA 3 and 4 designation was higher but did not reach significance (75.12% vs. 68.72%, *p* = 0.08). In the post-intervention group, the comparison showed that a higher number of ASA 3 and 4 patients obtained a PEC visit (98.25% vs. 76.20%, *p* < 0.001). Furthermore, a higher proportion of ASA 3 and 4 patients received PEC visits in the post-intervention group than in the pre-intervention group (98.25% vs. 75.12%, *p* < 0.001). The difference remained non-significant when the ASA status was adjusted as a covariate in the multiple logistic regression (OR = 1.2, 95% CI 0.96–1.50, *p* = 0.11).

## 4. Discussion

Our study demonstrated that an integrated telehealth PEP model was successful in reducing the number of patients requiring in-person PEC visits without any adverse effect on select perioperative quality of care outcomes. An overall reduction in PEC visits improved patient selection efficiency and reduced the cost burden associated with preoperative testing. The integrated telehealth visit proved noninferior to traditional PEC models for DOS testing, case cancellations, and case start delays. There are several reasons the DOS cancellation and first-case start delay rates may not have changed between the two groups. The cancellation rate at our institution was lower compared to those in other published studies [1,2]. Cancellations are also multi-factorial and can be related to administrative problems as well as patient variables [28,29]. Most patients (76%) in the pre-intervention group received an in-person PEC visit, which should have deemed them appropriately prepared for surgery. Our intervention did not involve the creation of a new PEC, as one already existed, but rather a new innovative approach to PEC screening that resulted in improved efficiency and likely cost due to decreased PEC visits. The IT optimizations allowed for an efficient and highly reliable method to streamline preoperative screening for a large population. We observed that the clinical decision support tool was the most effective IT-related intervention and its use likely resulted in less variability and errors compared to traditional paper formats [30]. The impact of the clinical decision support tool used in our IT-optimized telehealth PEC model is a prospective area for future research. DOS testing did not change between the pre-intervention and post-intervention groups, although there was a trend toward decreased DOS testing during the post-intervention period.

When individual DOS tests were analyzed, there was a greater amount of blood type and screen testing during the pre-intervention period compared to the post-intervention period. This decrease in the type and screen test volume during the post-intervention period was most likely related to the introduction of new institutional preoperative testing guidelines during the study. Furthermore, we found that there was a significant increase in the number of DOS electrocardiograms (ECGs) in the post-intervention time period. We suspect that the shift to “virtual” preoperative assessments made it more challenging to find a location for the patient to obtain a preoperative ECG. Although phlebotomy stations are readily available, the same does not exist in our area for ECGs. Furthermore, many primary care physician offices were not willing to perform an ECG on patients without a preoperative visit. To address this problem during the study period, we adapted hospital phlebotomy stations with the requisite equipment to perform ECGs.

A telehealth PEC model for preoperative anesthesia evaluation was a successful model in bariatric surgery patients and should be included in the bariatric care pathway. Although this study’s population consisted of bariatric surgical patients, its findings may be able to be extrapolated to other populations undergoing elective, intermediate-risk surgical procedures, while taking into consideration the significant variability in surgical populations when extrapolating findings from one population group to others.

### 4.1. Strength of the Study

The strengths of this study are its clinical relevance, clear methodology, and focused objectives. The study implemented interventions that represented ongoing trends in the PEP of surgical patients. The study was a multidisciplinary clinical study that addressed the clinical and administrative challenges of meeting the increased need for appropriate presurgical evaluation of a growing surgical population. The study demonstrated the clinical utility of utilizing the IT aspects of HER to create clinically feasible and customizable clinical pathways for specific surgical populations. The study’s population of bariatric surgery patients represents a population that typically harbors several comorbidities of moderate severity undergoing moderately invasive surgery. This patient population typically undergoes extensive presurgical evaluation and optimization. Therefore, the findings regarding the presurgical evaluation of this population are applicable to other patient populations that have typically moderate comorbidities and are in need of multidisciplinary presurgical evaluation and optimization in preparation for moderately invasive surgery. The before/after aspect of the study conducted within a single healthcare system of multiple institutions allows for the comparison of mostly uniform groups except for the element of the implemented intervention. Additionally, the retrospective examination of the study’s cohort allowed us to examine not only our primary outcome but also several secondary outcomes and the potential additional or reduced recourses required to implement the studied intervention. Finally, an appropriate and robust statistical analysis was implemented to filter out, to the extent possible, any potential confounders when drawing causal inferences between the study’s interventions and outcomes.

### 4.2. Limitations of the Study

Our study has several limitations. First, our regression analysis was adjusted only for the ASA physical status of the patient. While the before/after study design in a single health system would have afforded a significant degree of uniformity within the study cohort, it is possible that unexamined variables constituted confounding and drawing causal inferences between interventions and outcomes. Third, our analysis of the cost and resources needed for the implementation was limited by the data available. Fourth, the analyses of the causes of cancellations, delays, and additional testing on the DOS were limited by the data that were inputted into the EHR.

### 4.3. Future Research Possibilities

Future research possibilities include the prospective evaluation of the efficacy of integrating various clinical decision support tools with different IT-related interventions on the reliability and predictive performance of IT-integrated models. Additional areas of research include evaluating the performance of IT-integrated models in various pre-operative surgical populations and in complex healthcare systems.

## 5. Conclusions

In a bariatric surgery population, the use of an IT-enhanced telehealth triage pathway in an integrated PEP improved efficiency, as indicated by reduced PEC visits, and had no negative impact on the quality of care, as measured by cancelation, delays, and additional testing on the DOS. The intervention, utilizing existing features of the utilized HER, was easy to implement and resulted in a likely reduction in healthcare costs. This reduction of in-person PEC visits is particularly attractive during periods that require reduced personal contacts, such as during the COVID-19 pandemic.

## Figures and Tables

**Table 1:** Demographic variables.

Demographic Variable	Pre-Intervention (n = 810)	Post-Intervention (n = 740)	
Patient age (years, SD)	44.54 (12.13)	44.75 (12.05)	0.72
ASA (number, %)			
1	5 (0.63%)	4 (0.54%)	0.015
2	209 (25.80%)	147 (19.86%)	
3	579 (71.48%)	580 (78.38%)	
4	17 (2.10%)	9 (1.22%)	
Surgical procedure (number, %)			
Gastric bypass/Roux-En-Y *	132 (16.30%)	129 (17.43%)	0.07
Gastric band procedures ^†^	149 (18.40%)	153 (20.68%)	
Longitudinal gastrectomy ^‡^	512 (63.21%)	453 (61.22%)	
Revision ^§^	17 (2.10%)	5 (0.68%)	

Two-sample *t*-test or Fisher’s exact test *p*-value is shown. Abbreviations: SD = standard deviation; ASA = American Society of Anesthesiologists Physical Status Classification; CPT = Current Procedural Terminology * CPT: 43644 ^†^ CPT: 43770–43774 ^‡^ CPT: 43775 ^§^ CPT: 43848.

**Table 2:** Comparison of outcome measurements between groups.

Primary Outcome	Pre-Intervention (n = 810)	Post-Intervention (n = 740)	*p*-Value
PEC in-person visits	615 (75.93%)	114 (15.41%)	<0.001
Secondary outcomes			
Cost per PEC encounter (USD (SD))	USD 50.24 (24.46)	USD 30.56 (21.30)	<0.001
DOS cancellations (number (%))	9 (1.11%)	10 (1.35%)	0.84
First-case start delays (number (%))	566 (69.88%)	545 (73.65%)	0.1
Day of surgery testing * (number (%))	Pre-Intervention (n = 82)	Post-Intervention (n = 83)	
Day of surgery testing (any type)	24 (29.27%)	16 (19.28%)	0.13
Complete blood count	1 (1.22%)	4 (4.82%)	0.37
Type and screen	24 (29.27%)	0 (0.00%)	<0.001
Basic metabolic panel	0 (0.00%)	2 (2.41%)	0.5
Serum potassium	1 (1.22%)	1 (1.20%)	1
Liver function panel	0 (0.00%)	1 (1.20%)	1
Prothrombin/international normalized ratio	0 (0.00%)	1 (1.20%)	1
Partial thromboplastin time	0 (0.00%)	1 (1.20%)	1
Electrocardiogram	2 (2.44%)	13 (15.66%)	0.003
Chest radiograph	0 (0.00%)	0 (0.00%)	1

Note: Two-sample *t*-test or Fisher’s exact test *p*-value is shown. Abbreviations: PEC = preoperative evaluation center; DOS = day of surgery * Procedures requested by the anesthesiologist on the day of surgery. A random selection of 82 pre-intervention and 83 post-intervention patients were selected for analysis.

**Table 3:** Adjusted outcome variables.

In-Person PEC Visit	OR (95% CI)	*p*-Value
Post-intervention vs. Pre-intervention	0.05 (0.04–0.07)	<0.001
ASA 3–4 vs. 1–2	2.01 (1.49–2.70)	<0.001
**First-case start surgery delay**		
Post-intervention vs. Pre-intervention	1.20 (0.96–1.50)	0.11
ASA 3–4 vs. 1–2	1.09 (0.84–1.41)	0.528
**Cost outcome**		
	**beta (standard error)**	***p*** **-value**
Post-intervention vs. Pre-intervention	−20.07 (1.16)	<0.001
ASA 3–4 vs. 1–2	6.59 (1.37)	<0.001

Abbreviations: PEC = preoperative evaluation center; ASA = American Society of Anesthesiologists Physical Status Classification; OR: odds ratio; CI: confidence interval.

**Table 4:** Pre-operative evaluation center visits by ASA classification.

	PEC Visit		
Pre-Intervention	No (n = 195)	Yes (n = 615)	*p*-Value
ASA classification (number (%))			
1 and 2	61 (31.28%)	153 (24.88%)	0.08
3 and 4	134 (68.72%)	462 (75.12%)	
**Post-intervention**	**No (n = 626)**	**Yes (n = 114)**	
ASA classification (number (%))			
1 and 2	149 (23.80%)	2 (1.75%)	<0.001
3 and 4	477 (76.20%)	112 (98.25%)	

Fisher’s exact *p*-value is reported. Abbreviations: ASA = American Society of Anesthesiologists Physical Status Classification; PEC = preoperative evaluation center.

## Data Availability

The data presented in this study are available on request from the corresponding author. The data are not publicly available due to privacy.

## Appendix B. Preoperative Evaluation Center (PEC) Triage Algorithm

The following algorithm can be used to decide whether a patient gets an in-person PEC visit or virtual NP review.

NP = nurse practitioner

1. Surgical category
  - Very low risk procedures never need an in-person visit (examples: cataract, skin wide local excision, breast biopsy, Esophagogastroduodenoscopy (EGD), colonoscopy)
  - High-risk procedures always get an in-person visit (examples: Intrathoracic surgeries, aortic or other major vascular procedures, open heart procedures, major intra-abdominal procedures (hepatic lobectomy, cystectomy, pelvic exoneration))
2. Patient demographics that warrant a in-person visit (Very low risk surgical procedures will get an NP review)
  - History of difficult intubation
  - MI within last 1 year
  - Cardiac stents within last 1 year
  - Chest pain/angina
  - History of heart transplant
  - Home oxygen use
  - Cirrhosis
  - History of liver transplant
  - History of lung transplant
  - Pulmonary hypertension
  - End stage renal disease on hemodialysis
3. Patient demographics that warrant a NP review
  - Malignant hyperthermia (personal of family history)
  - Congestive heart failure
  - Pacemaker/Implantable cardiac defibrillator (ICD) (to review interrogation and recommendations)
  - Congenital heart defect
  - Muscular dystrophy
  - Cerebral palsy
  - Seizure within the last year
  - Stroke or transient ischemic attack (TIA) within the last year
  - Brain aneurysm
  - Intracranial tumor
  - Myasthenia gravis
  - Muscular dystrophy
  - Amyotrophic lateral sclerosis (ALS)/Lou Gehrig’s disease
  - Paralysis
  - Dyspnea/Shortness of breath
  - Bone marrow/stem cell transplant
  - Blood restrictions
  - History of head and neck radiation
  - History of trachesotomy
  - Chronic renal insufficiency
  - History of kidney transplant
  - Hyperthyroidism
  - Cocaine use (past or present)
4. Other patient demographics – If patient has 2–3 of the following, a NP review should be initiated; If patient has 4 or more, an in-person visit should be scheduled.
  - Anesthesia history
    - Post-operative nausea or vomiting (PONV)
    - Pseudocholinesterase deficiency (personal of family history)
  - Cardiovascular
    - Arrhythmia
    - Coronary artery disease
    - Myocardial infarction (MI)
    - Open heart surgery (coronary artery bypass graft (CABG) or valve surgery)
    - Cardiac stent (history of cardiac surgery)
    - Valve problem/murmur
    - Peripheral vascular disease
    - Deep vein thrombosis (DVT) or pulmonary embolus (PE)
  - Liver
    - Hepatitis B
    - Hepatitis C
    - Liver disease
  - Skin
    - Lupus
    - Scleroderma
  - Neuro
    - Stroke/ transient ischemic attack (TIA)
    - Nerve stimulator
  - Respiratory
    - Asthma/emphysema/COPD/chronic bronchitis
    - Obstructive sleep apnea
  - Hematology
    - Sickle cell disease
    - Bleeding disorder
  - Head and neck
    - Limited neck extension
    - Limited mouth opening
  - Endocrine
    - Diabetes on insulin
  - Other
    - Chronic pain/methadone/suboxone
    - Human immunodeficiency virus (HIV)/Acquired immunodeficiency syndrome (AIDS)
    - Steroid use in last 1 year
    - Hospitalization within the last 1 year
    - Age ≥ 80 years old
    - Body mass index (BMI) ≥ 40
    - Functional status < 4 METs (unable to climb 2 flights of stairs)
    - Patient on blood thinners/anti-coagulants (except aspirin)

Note: For surgeries scheduled within 5 days of their surgery date, an NP review will become a PAT visit.
